# Multi-omics integration provides biological insight and prioritizes potential drug targets in multiple sclerosis progression

**DOI:** 10.1186/s12974-026-03895-z

**Published:** 2026-06-02

**Authors:** Yuan Jiang, Jinyu Xiao, Ingrid Kockum, Pernilla Stridh, Qianwen Liu, Tomas Olsson, Lars Alfredsson, Xia Jiang

**Affiliations:** 1 Department of Clinical Neuroscience, The Karolinska Neuroimmunology and Multiple Sclerosis Centre, Centre for Molecular Medicine, Karolinska Institutet, Stockholm, 171 77 Sweden; 2 Institute of Environmental Medicine, Karolinska Institutet, Stockholm, 17177 Sweden; 3 Department of Epidemiology and Health Statistics, West China School of Public Health and West China Fourth Hospital, Sichuan University, 610041 Chengdu, China

**Keywords:** Progressive multiple sclerosis, Protein, Drug targets, Omics

## Abstract

**Introduction:**

Current therapies for multiple sclerosis (MS) primarily reduce relapse rates and delay disability by targeting inflammation, while have limited efficacy against disease progression driven by neurodegenerative processes. We sought to identify and validate proteins for MS progression by integrating a large genome-wide association study (GWAS) of MS progression with large-scale protein quantitative trait loci data from blood and brain.

**Methods:**

We conducted proteome-wide association studies (PWAS) to nominate proteins; applied summary-data Mendelian randomization and colocalization to evaluate association; and performed functional annotation (pathway enrichment, drug-target mapping, and protein-protein interaction networks) to prioritize therapeutic potential. Additionally, we performed external validation through bulk and cell-type-specific expression analyses and prioritized protein evaluation. The final key proteins were determined by triangulating evidence across all these streams.

**Results:**

We identified 48 genetically prioritized proteins. Functional annotation prioritized 14 with therapeutic potential and highlighted 13 non-MS drugs for repurposing. Triangulation of evidence with multi-omics external validation highlighted six key proteins: RRM2B (a *Cladribine* target), CBR1, and ETFA, which are linked to existing drugs; DNM3 (a GWAS-implicated locus), CAB39L, and NMRAL1, which emerged as validated novel proteins providing biological insight into MS progression.

**Conclusion:**

Our multi-omics integration prioritizes proteins implicated in MS progression, providing mechanistic insights into neurodegeneration and a foundation for future therapeutic exploration in progressive MS.

**Supplementary Information:**

The online version contains supplementary material available at 10.1186/s12974-026-03895-z.

## Background

Multiple sclerosis (MS) is a chronic, immune-mediated central nervous system (CNS) disease characterized by inflammation, demyelination, and neurodegeneration, leading to non-traumatic disabilities in millions of individuals worldwide [1]. Currently, over 20 approved disease-modifying therapies (DMTs) are available for treating MS, playing a crucial role in reducing relapse rates and delaying disability accumulation [1–3]. However, most existing therapies focus on mitigating neuroinflammation, which predominates in the early stage of MS, and have limited efficacy against the distinct neurodegenerative processes that drive disease progression [4]. Inadequate management of neurodegeneration symptoms may lead to treatment discontinuation and further worsen the disease trajectory [5]. Crucially, even in patients receiving therapies that effectively suppress relapses, there is often continued progression independent of relapse activity, a phenomenon largely attributed to underlying neurodegeneration and associated brain atrophy [6, 7]. These limitations stress an urgent need for continued efforts in drug discovery, development, and repurposing to better address MS development.

Compared to genes and transcripts, proteins, as the final products of genome expression, pose a more immediate and pronounced impact on phenotypes. Due to their pivotal roles in cellular and biological processes, proteins are regarded as highly effective drug targets. Numerous proteins, mostly involved in inflammation pathways, have been implicated in the pathogenesis of MS [8–12]. For example, using a highly sensitive proteomic immunoassay, a study identified 12 inflammation-related proteins (10 from cerebrospinal fluid (CSF) and two from plasma) associated with MS [11]. Building on this, a study partly verified these findings while further identified a combination of 10 CSF proteins effectively predicting the severity of disability worsening in MS [12]. These results, despite providing valuable insights into MS-linked proteins, are largely constrained by relatively small sample sizes, typically involving only a couple of hundred participants, leading to reduced statistical power and inconsistencies in protein identification [9–11]. As an alternative strategy, integrating large-scale proteomic data with comprehensive genetic information through computational biology approaches offers a promising strategy for effective identification, optimization, and prioritization of MS-associated protein candidates.

Through integrative strategies, six studies have prioritized several promising protein targets for MS, such as FCRL3, TYMP, AHSG, CD40, TNFRSF1A, CD58, PLEK, CR1, CD59, HLA-B, and TRAF3 [12–18]. However, a potential “pitfall” of these studies lies in their reliance on GWAS data related to MS susceptibility rather than progression [19]. Consequently, the identified proteins predominantly reflect early inflammatory activity [20], while markers of neurodegeneration remain largely overlooked [21]. This gap may partly explain the currently limited therapies in halting MS progression. Moreover, the limited availability of high-quality brain tissue datasets has led to the brain itself, a primary site of pathology, being significantly under-investigated, restricting a direct understanding of the CNS-specific mechanisms.

**Fig. 1:**
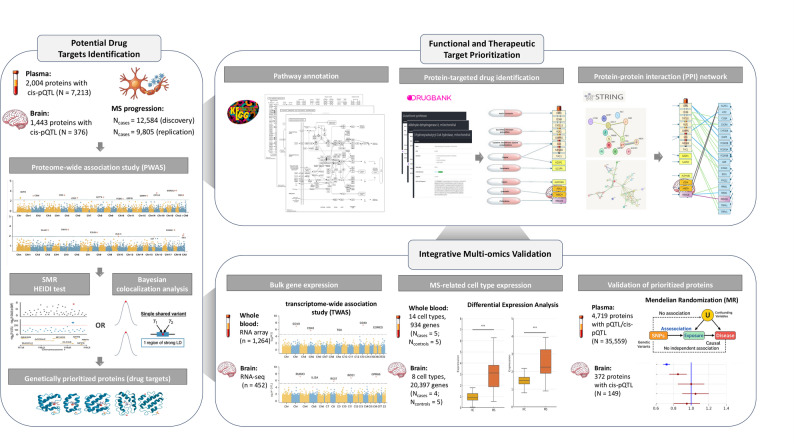
Flowchart of the overall study design. The study employed a multi-stage analytical framework to identify and validate protein targets for multiple sclerosis (MS) progression. First, a proteome-wide association study (PWAS) was performed to identify proteins associated with MS progression. These candidates were then filtered using summary-data-based Mendelian randomization (SMR) with the HEIDI test and Bayesian colocalization, yielding a set of genetically prioritized proteins. This set was subsequently subjected to two parallel analyses: a functional and therapeutic target prioritization (pathway analysis, protein-protein interaction, and drug-target identification) and multi-omics external validation (Mendelian randomization, transcriptome-wide association study, and differential expression analysis). Finally, evidence from all stages was integrated to identify the final key protein targets

Leveraging a recent large genome-wide association study (GWAS) on MS progression [21], our study aimed to prioritize drug targets for mitigating MS progression by integrating this data with the hitherto largest available summary statistics of protein quantitative trait loci (pQTL) [22, 23]. We investigated protein expressions not only in plasma, an easily accessible material reflecting peripheral protein levels, but also in brain, the primary site of MS pathology, where proteins play crucial roles in immune regulation, myelin repair, neuroinflammation, and neurodegeneration. We employed proteome-wide association studies (PWAS), summary-data-based Mendelian randomization (SMR), and colocalization analysis under an integrative framework to identify genetically prioritized proteins. Furthermore, we annotated the implicated proteins with biological pathways, investigated their protein-protein interactions (PPI) with known MS drug targets to prioritize the proteins with therapeutic value, as well as to explore repurposing opportunities. Finally, utilizing external data, we performed Mendelian randomization (MR), transcriptome-wide association study (TWAS), and single-cell RNA-seq differential expression analysis (DEA) to validate the biological relevance of the prioritized proteins (Fig. 1). Through this integrative framework, we identified and validated a set of high-confidence proteins that represent promising new targets for therapies aimed at mitigating MS progression.

## Methods

### Plasma and brain pQTL summary statistics

Plasma pQTL summary statistics were sourced from a cohort study including 7,213 participants of European ancestry [23]. Plasma protein levels were quantified using modified aptamer-based technology (SOMAmer). After quality control, 4,657 SOMAmers with tagged proteins encoded by 4,435 genes were identified. Genotyping data were generated using the Affymetrix 6.0 DNA microarray and imputed with the TOPMed reference panel. Post-imputation quality control steps included filtering variants based on imputation accuracy, Hardy-Weinberg equilibrium (HWE), and minor allele frequency (MAF), resulting in 6,181,856 SNPs. pQTLs were identified by applying linear regression models, adjusting for age, sex, study site, and the ten genetic principal components (PCs). Cis-regions were defined as 500 KB upstream and downstream of transcription start sites. This analysis determined 2,004 proteins with significant cis-pQTL associations.

Brain pQTL summary statistics were derived from two clinical-pathologic cohort studies using the dorsolateral prefrontal cortex of postmortem brain samples donated by 400 participants of European ancestry [22]. Brain protein levels were quantified using liquid chromatography and mass spectrometry. Quality control procedures included filtering for outliers, missing data, and protein loading values. A total of 8,356 proteins in the brain were successfully quantified. Genotyping was conducted using either whole-genome sequencing or genome-wide genotyping platforms (Illumina OmniQuad Express and Affymetrix GeneChip). Imputation was performed based on the 1000 Genomes (1KG) reference panel. Quality control of SNPs included checks for missingness, HWE, imputation quality, MAF, and relatedness. After these steps, 1,190,321 SNPs on autosomal chromosomes that aligned with the HapMap linkage disequilibrium reference panel were retained. Linear regression models were used to identify pQTLs. Cis-regions were defined as 500 KB upstream and downstream of genes. In total, 1,443 significant proteins with cis-pQTL were identified.

### GWAS of MS progression

Summary statistics of MS progression were derived from the latest GWAS, where severity was assessed using the age-related MS severity score (ARMSS) [21]. The study included 12,584 MS cases of European ancestry as a discovery cohort, of which results were replicated in a further cohort of 9,805 cases. The discovery cohort comprised MS patients recruited from 21 centers, genotyped on a common platform (Illumina Global Screening Array). The replication cohort included MS patients with clinical data from nine European centers, genotyped using various Illumina arrays. Quality control of population and SNP was performed, including checks on MAF, genotype missingness, relatedness, and non-palindromic variants. Imputation was performed using the Haplotype Reference Consortium panel (release 1.1) via Minimac4. Covariates in the GWAS model included age, sex, date of birth, expanded disability status scale (EDSS) source (neurologist assessment versus questionnaire), center, genotyping batch, and the first ten PCs.

All genomic data used in this study were processed and aligned to the hg19 human reference genome. Summary statistics and genomic sources for all public GWAS datasets are listed in Table S1.

### Statistical analysis

#### PWAS identifying candidate proteins associated with MS progression by integrating pQTL and GWAS of MS progression

To identify proteins potentially associated with MS progression, a PWAS analysis was conducted. FUSION software and pre-computed elastic net-based weights for protein expression in both plasma and brain tissues were used [22, 23]. Firstly, the 1KG reference panel was employed to reduce the impact of LD effects on the MS progression GWAS. Then, SNP effect sizes (z-scores) from GWAS were imputed using the ImpG-Summary algorithm [24]. Finally, a linear regression model was applied to estimate the association between protein weights and GWAS results of MS progression. To maximize potential proteins discovery, PWAS was implemented as an exploratory screening step. Proteins associated with MS progression at a nominal threshold of *P* < 0.05 were carried forward as candidates for downstream evaluation. Candidate proteins were subsequently prioritized using SMR with the HEIDI test, Bayesian colocalization, and independent multi-omics validation. The Bonferroni-corrected threshold was also indicated in the PWAS figures for transparency.

#### SMR analysis for protein prioritization

To further evaluate if the proteins identified through PWAS played a role in MS progression, SMR analysis was performed [25]. The SMR method evaluates the pleiotropic association between genetically predicted protein levels and MS progression, under the assumption that a single causal variant influences both protein expression and disease phenotype. However, due to LD, the SMR effect may be wrongly estimated even if the underlying assumption is not violated. To address this, the Heterogeneity in Dependent Instruments (HEIDI) test was conducted. HEIDI test assumes that if a shared causal variant drives both traits, the β_SMR_ values should remain consistent across variants in LD. Significant heterogeneity in β_SMR_ across cis-pQTLs would thus suggest linkage rather than pleiotropy. Both SMR and HEIDI analyses were conducted using the SMR software (version 1.3.1). Standard quality control procedures were first performed, including exclusion of variants with MAF < 0.01 (--maf 0.01) and removal of SNPs exhibiting substantial allele frequency discrepancies between the GWAS and pQTL datasets (--diff-freq 0.2), to reduce potential strand ambiguity. The 1KG reference panel was used for allele alignment. For the SMR test, pQTLs with a *P*-value < 5 × 10^− 8^ were included in the analysis. For the HEIDI test, pQTLs were required to have an *R*² of 0.05–0.90 with the top cis-pQTL, a *P*-value < 1.57 × 10^− 3^, and a total number of SNPs between 3 and 20. To maximize the sensitivity of candidate discovery for this complex trait, proteins passing both the SMR test (*P*_SMR_ < 0.05 and an exploratory threshold of FDR-adjusted *P*_SMR_ < 0.10 [26]) and the HEIDI test (*P*_HEIDI_ > 0.05) were defined as genetically prioritized proteins.

To mitigate potential measurement artifacts in aptamer-based assays, we annotated the top cis-pQTLs for all prioritized proteins to identify overlaps with known protein-altering variants (PAVs). For plasma proteins (aptamer-based), if the primary instrument was a PAV, a sensitivity SMR analysis was performed by excluding the PAV. For brain proteins, which were quantified using Mass Spectrometry, the risk of epitope-binding artifacts is inherently minimized. Therefore, the sensitivity analysis excluding PAVs was not required.

#### Bayesian colocalization analysis for shared genetic architecture

To further address the effects of LD on the potential pleiotropic associations, a Bayesian co-localization analysis was performed using the coloc R package (version 5.2.3). This method evaluates the posterior probabilities for five hypotheses, H0: neither protein expression nor MS progression is genetically associated in the region; H1: only protein expression is genetically associated; H2: only MS progression is genetically associated; H3: both traits have genetic associations but are influenced by different causal variants; H4: both traits are genetically associated and share a single causal variant. A posterior probability for H4 (PPH4) > 0.50 was considered indicative of colocalization [27]. To assess the robustness of colocalization findings, we performed sensitivity analyses across varied prior configurations. To further support a shared genetic architecture rather than independent genetic effects, we generated regional association plots for the MS severity GWAS and the corresponding cis-pQTLs within ± 500 kb of the lead variant. Summary-level conditional analyses were performed by conditioning both GWAS and pQTL associations on the lead variant. Attenuation of the GWAS signal after conditioning, together with loss of the pQTL association, was used as supportive evidence for a shared underlying genetic signal.

#### Definition of genetically prioritized proteins and key proteins

We defined the PWAS-identified proteins that passed either SMR (*P*_SMR_ < 0.05, FDR-adjusted *P*_SMR_ < 0.10, and *P*_HEIDI_ > 0.05) or co-localization (PPH4 > 0.50) as genetically prioritized proteins of MS progression. This initial set of proteins was then subjected to two complementary analytical strategies. First, a functional annotation analysis (KEGG, PPI, and DrugBank) was performed to identify a set of prioritized proteins based on their therapeutic relevance and opportunity for drug repurposing. Second, a multi-omics validation analysis (MR, TWAS, and DEA) was performed to confirm the biological robustness. The final key proteins were then selected by integrating evidence from all streams of our study.

#### KEGG pathway annotation, drug target identification, and PPI network analysis

To explore the functional relevance of the identified prioritized proteins, pathway annotation was performed using the KEGG pathway database (https://www.genome.jp/kegg/pathway.html). Drugs targeting the identified proteins, along with existing MS drug targets, were identified by referencing the DrugBank database (https://go.drugbank.com/). Protein-protein interaction (PPI) network analysis was conducted using the STRING database (https://stringdb.org/) to examine interactions among the identified drug targets and known MS drug targets. Only interactions with a confidence score above 0.70 were shown.

#### Validating proteins using causal evidence from MR

Although SMR with the HEIDI test and colocalization analysis can effectively reduce the impact of LD, distinguishing between vertical pleiotropy (causality) and horizontal pleiotropy remains challenging. To further strengthen our findings, we conducted MR analysis using external pQTL data. Specifically, we utilized summary statistics from a large plasma pQTL study (*N* = 35,559) covering 4,719 proteins, as well as summary statistics from a brain cis-pQTL study (*N* = 149) covering 372 proteins [28, 29]. First, we performed a genome-wide MR using instrumental variables (IVs) across the genome. Second, recognizing that trans-acting pQTLs could introduce pleiotropy via indirect biological pathways, we conducted a more conservative cis-MR, restricting IVs to the cis-region defined as the genomic window ± 500 kb from the transcription start site of genes encoding the proteins of interest. To select the independent IVs, we first filtered variants from the summary statistics for genome-wide significance (*P*-value < 5 × 10^− 8^) and subsequently clumped them with an *r*^*2*^ threshold of 0.10 within a ± 500 KB window. Allele harmonization was performed using TwoSampleMR (harmonise_data, action = 1) because effect allele frequency was unavailable in some datasets. As forward-strand allele coding was ensured by the original cohort processing/imputation pipelines, harmonization was conducted under the forward-strand assumption. For the genetically prioritized plasma proteins identified by our main results, genome-wide and cis-pQTL data were available for 23 and 18, respectively. For brain proteins, only cis-pQTL data were available for nine proteins.

The inverse variance weighted (IVW) approach was performed. Additionally, the MR-Egger intercept test was used to reflect directional pleiotropy. For proteins incorporating only one independent instrument, Wald ratio and robust adjusted profile score (MR-RAPS) were applied. To evaluate instrument strength, we calculated the variance explained (*R*^*2*^) from the exposure effect size and standard error, and derived the corresponding *F*-statistic to evaluate the risk of weak instrument bias. All analyses were conducted with packages “TwoSampleMR”, “MRInstruments”, “MendelianRandomization”, and “mr.raps” in R v3.6.3. Significance was defined as a *P*-value < 0.05. For MR using a single IV, consistency in the direction of effect was required across the Wald ratio and the MR-RAPS. MR-Egger intercept with a *P*-value < 0.05 was used to indicate directional pleiotropy.

#### Validating proteins using predicted gene expression from TWAS

To investigate the mRNA expression levels of the identified proteins in gaining biological insight, TWAS analysis was performed using the FUSION software. Pre-computed expression reference weights were obtained from the FUSION database, including RNA array data from whole blood (*n* = 1,264) and RNA-seq data from brain tissue (*n* = 452). The significance threshold was set at *P*-value < 0.05. In total, this analysis covered 13 genetically prioritized proteins from plasma and four from brain.

#### Validating proteins using cell-type specific expression from DEA

To investigate whether the genes encoding proteins are expressed in MS-related cell types, we checked DEA between MS patients and controls in single-cell transcriptomes. For plasma proteins, single-cell transcriptomics data from whole blood samples of five MS patients and five controls were utilized [30]. DEA was originally performed in this study across 14 blood cell types using the Bayes factor method and the EdgeR method. For brain proteins, we utilized single-nucleus RNA sequencing data from the white matter area in the brains of four progressive MS patients and five controls [31]. After quality control and normalization, we performed DEA across eight brain cell types using the Wilcoxon Rank Sum test. Multiple testing correction was applied using the FDR, adjusting for all genes within the specific cell type, with a significance threshold set at FDR-adjusted *P* < 0.05. To ensure biologically meaningful results, we applied an absolute average log2 fold change > 0.10, indicating at least a 10% differential expression of a gene in the target cell type compared to the reference group. Only genes exhibiting significant differential expression with a consistent direction of effect at both transcriptomic and proteomic levels were retained. In total, this analysis covered 16 genetically prioritized proteins from plasma and 10 from brain.

## Results

### Identifying candidate proteins associated with MS progression through PWAS

**Fig. 2:**
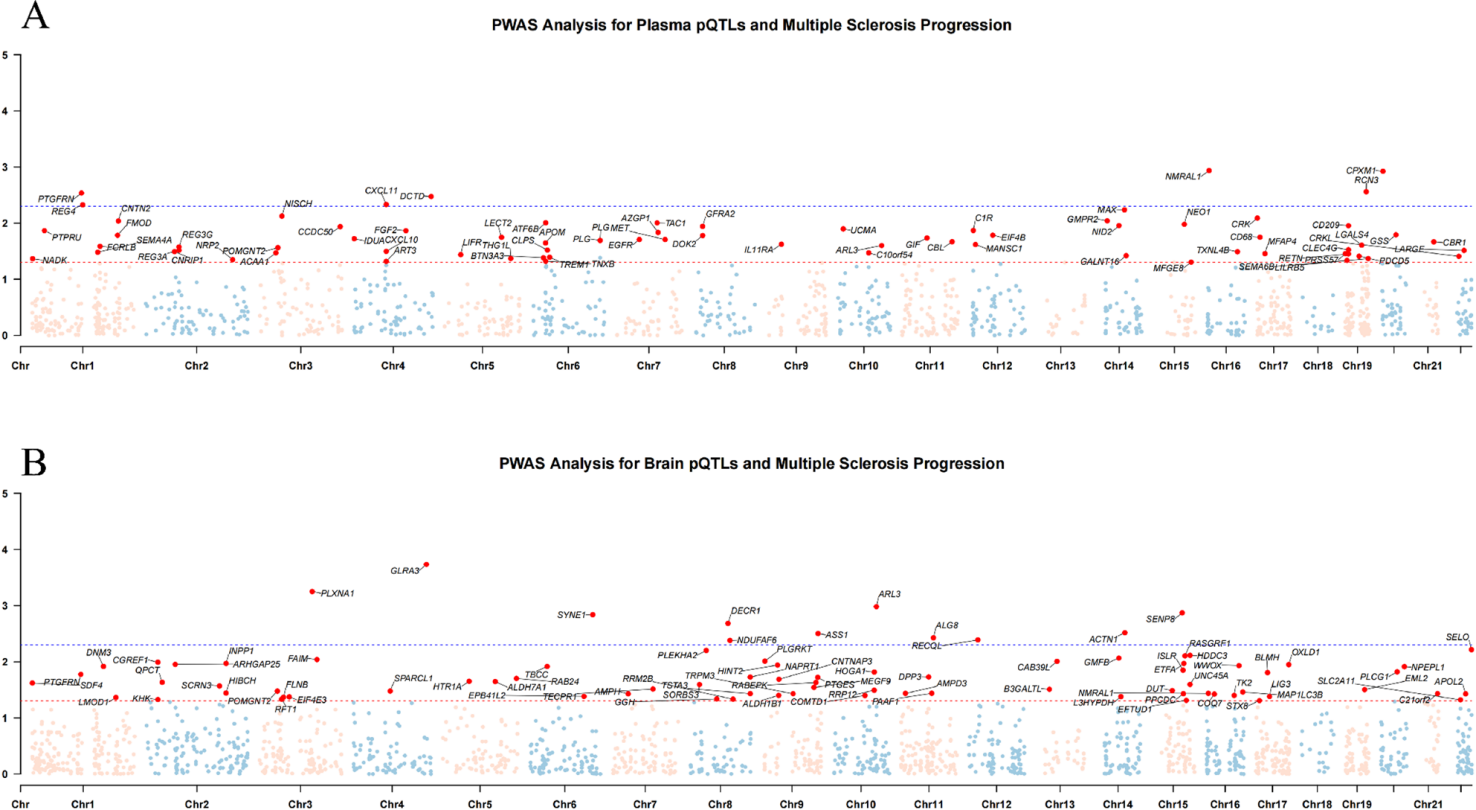
Identifying candidate proteins for MS progression by integrating pQTL and GWAS through PWAS. Manhattan plots showing the results of the proteome-wide association study (PWAS) in (**A**) plasma and (**B**) brain. The y-axis represents the -log10 (*P*-value) for the association of each protein with multiple sclerosis (MS) progression, plotted against its chromosomal position (x-axis). The red dashed line indicates the significance threshold (*P* = 0.05). The blue dashed line indicates Bonferroni corrected significance threshold. Proteins exceeding this threshold are identified as candidate proteins and highlighted in red. pQTL: protein quantitative trait loci; GWAS: genome-wide association study; PWAS: proteome-wide association study; Chr: chromosome

To identify candidate proteins linked to MS progression, we employed PWAS to establish associations between protein levels in plasma and brain and MS progression. In plasma, we identified 72 proteins whose expression levels were associated with MS progression (*P*-values < 0.05). Correspondingly, in the brain, we identified 83 proteins (*P*-values < 0.05). The 72 plasma proteins and 83 brain proteins (Fig. 2; Table S2 & S3) were considered as candidates for the subsequent analysis.

### Identifying genetically prioritized proteins associated with MS progression through SMR and colocalization

We employed SMR to investigate genetically prioritized proteins identified through PWAS. In plasma, we identified 29 proteins whose expression levels were associated with MS progression (*P*_SMR_ < 0.05, FDR-adjusted *P*_SMR_ < 0.10, and *P*_HEIDI_ > 0.05, Table S4). Colocalization analysis identified a single protein, CNTN2 (PPH4 > 0.5), a known autoantigen in MS patients that has been implicated in gray matter pathology [32] (Table S5). In total, we identified 30 genetically implicated proteins in the plasma (Table S8).

In brain tissue, 19 proteins were found to have expression levels associated with MS progression (*P*_SMR_ < 0.05, FDR-adjusted *P*_SMR_ < 0.10, and *P*_HEIDI_ > 0.05). Among these, CAB39L was particularly noteworthy, as it was the only candidate that also showed evidence of colocalization (PPH4 > 0.5) (Table S6 & S7). Moreover, our identification of DNM3 represented a crucial validation, as it strengthened a previously suggestive association of rs149097173 in the *DNM3*-*PIGC* locus from the MS progression GWAS [21] by providing evidence of a link at the protein level. In total, we identified 19 potential causative proteins in the brain (Table S8).

Sensitivity analyses excluding identified PAVs validated the robustness of our SMR estimates, with associations remaining consistent across plasma datasets (Table S9). Sensitivity analysis using varied prior configurations demonstrated that our colocalization results remained robust, with PPH4 values remaining stable across most tested parameter settings (Table S10 &S11). Notably, NMRAL1 was identified in both plasma and brain through SMR analysis, suggesting it may play a systemic role in MS progression. In total, we identified 48 genetically prioritized proteins in plasma and brain that influenced MS progression (Table S8).

### Pathway annotation, protein-targeted drugs identification, and PPI to prioritize proteins with therapeutic value

To understand the biological mechanisms of the 48 prioritized proteins and identify drug repurposing opportunities, we conducted functional analyses including pathway annotation, PPI analyses, and protein-targeted drug identification.

**Fig. 3:**
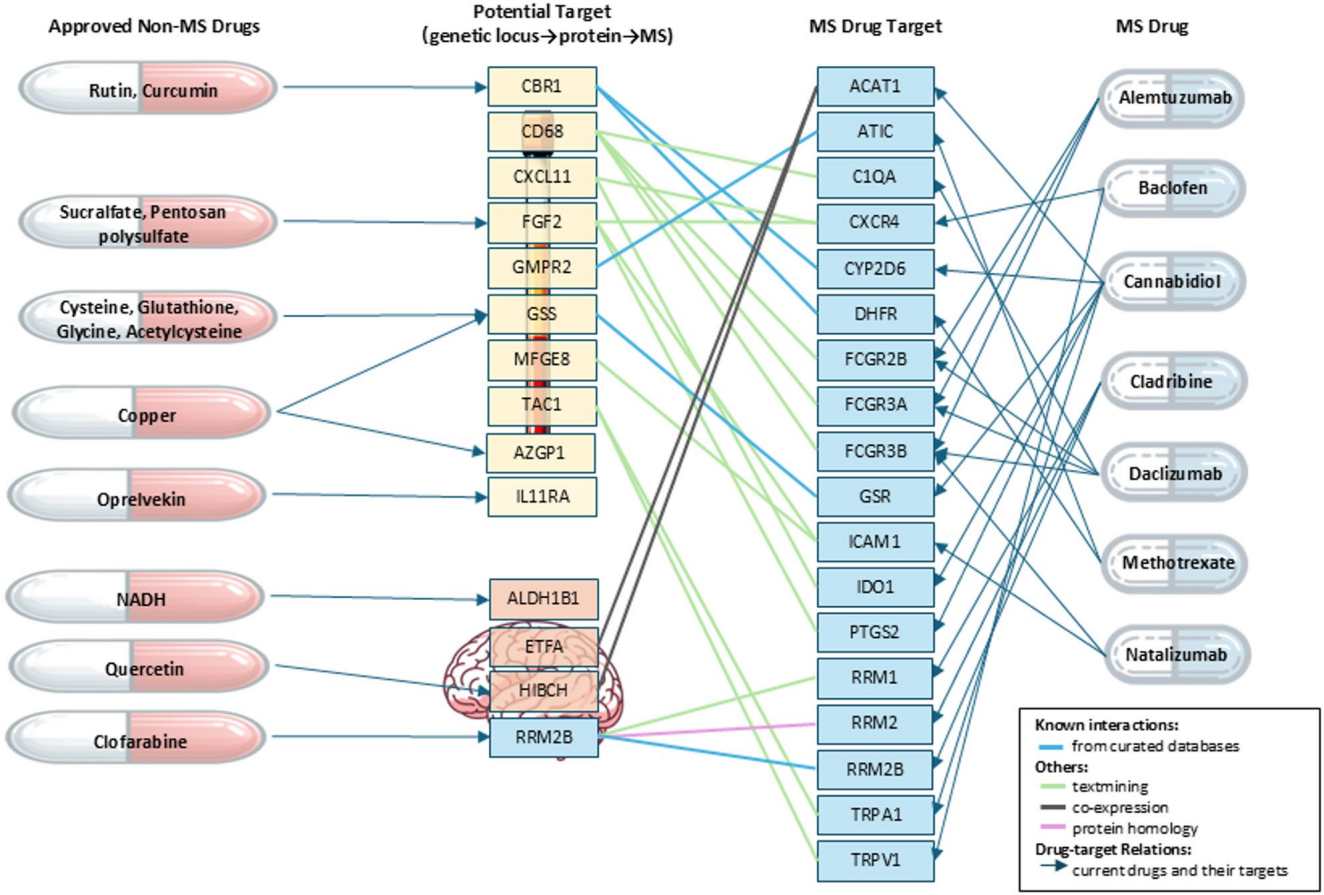
Protein-protein interaction network and drug-target relationships for genetically prioritized proteins. The network illustrates interactions between genetically prioritized proteins (labeled by their encoding gene names) and known targets of existing multiple sclerosis (MS) drugs. Proteins identified in plasma are shown in yellow boxes, while those from the brain are in orange. Proteins that are themselves targets of current MS drugs are highlighted with purple boxes. Lines of different colors represent various types of interaction evidence from the STRING database. Approved drugs for non-MS indications that target the identified proteins are also shown

For the 30 proteins detected in plasma, 62 pathways were annotated (Table S12). Metabolic pathways were the most frequently linked, marked by five proteins. In PPI analysis (Fig. 3; Table S14), eight MS progression-related proteins (GMPR2, CD68, FGF2, CXCL11, CBR1, GSS, MFGE8, and TAC1) were found to interact with 14 known drug targets of six existing MS therapies (*Methotrexate*, *Daclizumab*, *Baclofen*, *Cannabidiol*, *Alemtuzumab*, and *Natalizumab*). Additionally, five identified proteins (CBR1, FGF2, GSS, AZGP1, and IL11RA) were targets of 10 non-MS drugs. Among these drugs, five (*Cysteine*, *Glutathione*, *Glycine*, *Copper*, and *Rutin*) are either nutrients or natural supplement products, presenting promising opportunities for drug repurposing.

For the 19 proteins detected in the brain, 32 pathways were annotated (Table S13), with metabolic pathways again being the most frequently implicated, involving four proteins. Interactions were observed between two MS progression-related proteins (ETFA and HIBCH) and ACAT1, the target of an existing MS drug, *Cannabidiol*. Furthermore, our analysis identified HIBCH and ALDH1B1 as candidates for drug repurposing, as they are the respective targets of the non-MS compounds *Quercetin* and *NADH*-both are already used in nutritional supplements. Notably, the identification of RRM2B is a key therapeutic finding because it is the target of two drugs: *Cladribine* and its sister drug, *Clofarabine*. This discovery directly links the established relapse therapy *Cladribine* to the mechanisms of neurodegeneration and identifies *Clofarabine* as a candidate warranting further experimental and clinical evaluation (Fig. 3; Table S14).

Collectively, these functional analyses revealed extensive therapeutic connections, prioritizing 11 proteins that interact with 17 targets of existing MS therapies and eight proteins targeted by 13 non-MS drugs, with the link between RRM2B and the approved MS drug *Cladribine* being the most notable.

### Integrative multi-omics validation pinpoints key proteins

We implemented an integrative validation strategy for the 48 genetically prioritized proteins identified in our initial screen. This involved assessing genetic causality, establishing mRNA association, and demonstrating specific dysregulations in disease-relevant cell types for all candidates for the subset of proteins covered by the respective external datasets (Table S15-S18).

In plasma, our integrative approach validated several key candidates. CBR1 stood out, with its causal role confirmed by MR and its expression significantly reduced in pathogenic immune cells like CD4 + T cells. In the brain, MR analysis validated the causal roles of RRM2B, ETFA, and NMRAL1, and evidence from TWAS further implicated CAB39L. Among the three candidate genes detected in the white matter single-cell RNA panel, CAB39L and RRM2B exhibited significantly lower levels of expression in three brain cell types (oligodendrocyte precursor cell, astrocyte, and neuron), while DNM3 exhibited higher expression in endothelial cells.

Taken together, this integrative validation supported the potential pathogenic roles of six key proteins (RRM2B, CBR1, ETFA, DNM3, CAB39L, and NMRAL1) and underscored their potential as therapeutic targets. Marginal and conditional regional association plots of six key proteins are presented in Figure S1-6, showing that conditioning on the lead variant markedly attenuated the GWAS associations and abolished the pQTL signals, supporting a shared regional genetic basis and complementing the Bayesian colocalization results.

## Discussion

In this study, we tested hundreds of MS-associated proteins in plasma and brain and genetically prioritized 48 proteins (30 in plasma and 19 in brain with one dual-significant protein) through comprehensive analytical strategies. Our analyses revealed that 11 prioritized proteins (CBR1, CD68, CXCL11, FGF2, GMPR2, GSS, MFGE8, and TAC1 in plasma; ETFA, HIBCH, and RRM2B in brain) were linked to 17 known MS drug targets, with the connection between RRM2B and an approved DMT, *Cladribine*, being the most direct therapeutic link. Furthermore, we identified potential drug repurposing opportunities, as seven prioritized proteins (CBR1, FGF2, GSS, AZGP1, and IL11RA in plasma; HIBCH, ALDH1B1, and RRM2B in brain) were targets of 13 existing non-MS drugs. External multi-omics validation prioritized six key proteins, including four brain-derived candidates (RRM2B, ETFA, DNM3, and CAB39L) that provide CNS-relevant biological insight but should be interpreted as exploratory signals requiring further validation in larger datasets and experimental studies. These six key proteins collectively point toward a range of novel therapeutic strategies for MS progression. The most direct opportunities are highlighted by RRM2B, the target of the approved MS drug *Cladribine*, alongside CBR1 and ETFA, which connect to other existing DMTs. Meanwhile, our findings provided key insight into the disease biology by validating the key GWAS locus DNM3, identifying NMRAL1 as a dual-tissue protein implicated in both central and peripheral pathogenesis, and highlighting CAB39L as the candidate with the strongest genetic evidence supported by both SMR and colocalization.

Among the prioritized proteins, RRM2B holds the most established connection to current MS therapies. It plays a pivotal role in repairing damaged DNA and preserving mitochondrial DNA integrity [33], a process known to be dysregulated in MS patients [34]. Its significance is underscored as it is the mechanistic target of two purine nucleoside analogs [35]: the approved MS therapy *Cladribine* [36, 37], and another drug *Clofarabine*. The connection to *Cladribine* is particularly notable. While this DMT is effective for relapsing-remitting MS (RRMS), its impact on the neurodegenerative aspects of secondary progressive MS (SPMS) appears to be limited-though this may be due to the existing limitations of prior studies such as small sample sizes [38]. Our identification of its target, RRM2B, specifically in the context of progression provides a genetically supported rationale to re-evaluate *Cladribine* in robustly designed trials focused on neurodegeneration. Furthermore, our discovery that RRM2B is also a target for *Clofarabine* highlights a completely unexplored but promising candidate for repurposing in MS progression. Further therapeutic relevance is highlighted by CBR1 and ETFA, as our analysis revealed that both proteins interact with targets of another existing DMT, *Cannabidiol*. This shared link to a therapeutic pathway strengthens their potential as therapeutic targets. Individually, CBR1 exerts neuroprotective effects by mitigating oxidative stress-a key driver of neurodegeneration in progressive MS [39]. Likewise, ETFA is integral to mitochondrial fatty acid β-oxidation [40], a pathway modulated by riboflavin [41], which has shown protective effects in MS models [42]. Taken together, these connections provide a strong rationale for formally investigating the roles of these proteins in MS progression, supporting their premise as high-priority candidates for functional validation.

Our findings contribute to the understanding of the complex nature of MS progression by identifying key proteins involved in multiple pathological processes. The validation of DNM3 is a crucial finding, as it further supports a key locus from the foundational MS progression GWAS [21] at the protein level. This finding is particularly significant given DNM3’s known biological roles in the CNS. Functionally, it participates in the morphogenesis of the postsynaptic density and in excitatory synaptic transmission [43]. Furthermore, its preferential expression in neurons and oligodendrocyte lineage cells [21] directly aligns with the known pathology of MS progression, as these cell types are critically implicated in neurodegeneration [44]. In addition to this key validation, our study highlights CAB39L for its potential link to symptom management. Its role in the mTOR signaling pathway [40], a key pain regulator [45], is highly relevant given a prior GWAS linking it to neuropathic pain [46], a frequent and debilitating MS symptom. Finally, NMRAL1 suggests a novel direction for research, as this innate immunity and redox-regulating protein has established genetic links to a brain disorder schizophrenia [47], but its function in MS progression has been entirely unexplored. Together, the genetic prioritization of DNM3 and the novel insights into CAB39L and NMRAL1 represent significant advances in understanding the biological drivers of MS progression and provide a foundation for developing novel therapeutic strategies.

Most patients with MS are initially diagnosed with a RRMS, but 50–60% eventually transit to SPMS, a phase marked by irreversible neurodegeneration and worsening disability [4, 48]. While current DMTs effectively target neuroinflammation in RRMS and reduce relapses, halting the distinct neurodegenerative mechanisms of SPMS remains a major therapeutic limitation. This is reflected in the limited therapeutic options for SPMS, where the few approved therapies, such as *Siponimod*, demonstrate only modest efficacy in slowing progression [38], while promising new agents like the BTK inhibitor *Tolebrutinib* are still in clinical trials [49]. Our findings address this critical therapeutic gap by providing a data-driven rationale for drug repurposing. As detailed for RRM2B, our work suggests a potential rationale to re-evaluate an established MS therapy-*Cladribine*-for a potential role in neuroprotection, and brought its sister drug, *Clofarabine*, into focus as a completely new candidate. Furthermore, our analysis revealed numerous repurposing opportunities among non-MS compounds, particularly seven readily available nutrients and supplements. The identification of targets for flavonoids like *Rutin* and *Quercetin* is especially promising, as their known protective effects against demyelination [50, 51] suggest their potential as adjunctive therapies. Collectively, these findings suggest several potential therapeutic strategies for MS progression, providing a rationale to re-evaluate existing drugs for novel adjunctive MS treatments.

The results of our analyses should be interpreted with caution. First, the initial PWAS used a nominal P-value threshold, which increased sensitivity but may also have introduced false-positive associations. This funnel-based strategy was chosen to avoid prematurely excluding potentially relevant proteins at the discovery stage, particularly given the limited GWAS power for MS progression. Indeed, only one of the six key proteins ultimately supported by downstream validation reached Bonferroni-corrected significance in the initial PWAS. Therefore, PWAS results were interpreted only as exploratory candidate-generating evidence, and downstream conclusions were based on proteins further supported by additional genetic and multi-omics analyses. Second, the statistical power disparity between the plasma (*N* = 7,213) and brain (*N* = 400) pQTL datasets must be noted. The smaller brain cohort inherently limits the detection of variants with subtle effects. Nevertheless, we prioritized the inclusion of brain proteomics because localized changes within the CNS are expected to more closely reflect the biological processes of MS progression. Given the difficulties in obtaining post-mortem CNS tissues, these data offer etiologically essential insights. We have therefore framed the brain-derived results as CNS-relevant exploratory signals rather than definitive causal evidence, which also require further validation. Third, not all proteins have pQTLs. In our referenced plasma pQTL study, only 45% (2,004 out of 4,435) of the proteins exhibited significant cis-pQTLs [23]. The dependence on pQTLs for integrative analysis may thus result in the exclusion of important proteins. This may explain, for instance, why the known targets of effective SPMS therapies like *Siponimod* did not emerge from our analysis, as these specific proteins may lack detectable pQTLs in the available datasets. This challenge is further exemplified by our follow-up of four key genes from a foundational MS progression GWAS [21], where pQTL data were only available for two (DYSF and DNM3). The successfully validation on one of these, DNM3, under these circumstances underscores the robustness of this finding. However, the insignificant result for protein DYSF introduces ambiguity regarding the true causal molecule at the *DYSF-ZNF638* genetic locus. It is likely that the genetic signal may instead exert its effect through the nearby ZNF638 protein, but this hypothesis could not be evaluated as protein level data for ZNF638 was not available in the referenced pQTL dataset. Such ambiguity underscores the need for larger and more comprehensive pQTL studies to identify the prioritized proteins. Finally, while our multi-omics approach provides a systematic framework for genetic and biological target prioritization, these findings remain inherently computational. The identified protein targets and drug-repurposing opportunities should therefore be interpreted as hypothesis-generating signals rather than definitive causal mechanisms or clinical recommendations. Similarly, drug-target links identified through DrugBank and PPI analyses indicate potential repurposing opportunities but do not establish efficacy, safety, or clinical applicability in MS progression. Rigorous functional studies are required to validate the biological roles of the prioritized proteins, confirm their therapeutic relevance in disease models, and evaluate whether modulation of these targets could have clinical utility in MS progression.

## Conclusions

In conclusion, our comprehensive analysis identified 48 genetically prioritized proteins in MS progression, from which 14 were prioritized for their high therapeutic value and 13 non-MS drugs were identified for repurposing. Combining evidence from multi-omics validation, we pinpointed six key proteins implicated by two key themes from our study, RRM2B (linked to the MS therapy *Cladribine*), CBR1, and ETFA underscore actionable therapeutic pathways, while DNM3, CAB39L, and NMRAL1 reveal novel pathogenic insights. Collectively, these findings provide the foundation for developing new strategies that eventually address MS progression.

## Supplementary Information

Supplementary Material 1.

Supplementary Material 2.

Supplementary Material 3.

Supplementary Material 4.

Supplementary Material 5.

Supplementary Material 6.

Supplementary Material 7.

## Data Availability

All the genetic and QTL data used in this study were from the publicly accessible summary statistics and can be accessed through the corresponding references presented in the main text.

## Abbreviations

ARMSS: Age-related MS severity score
CNS: Central nervous system
CSF: Cerebrospinal fluid
DEA: Differential expression analysis
DMTs: Disease-modifying therapies
EDSS: Expanded disability status scale
eQTL: Expression quantitative trait loci
GWAS: Genome-wide association study
HEIDI: Heterogeneity in dependent instruments
HWE: Hardy-Weinberg equilibrium
IVW: Inverse variance weighted
MAF: Minor allele frequency
MR: Mendelian randomization
MS: Multiple sclerosis
PCs: Principal components
PPI: Protein-protein interactions
PPH4: Posterior probability for H4
pQTL: Protein quantitative trait loci
PWAS: Proteome-wide association studies
SMR: Summary-data-based Mendelian randomization
TWAS: Transcriptome-wide association study
