# Supplementary figures and images for: Multi-omics integration provides biological insight and prioritizes potential drug targets in multiple sclerosis progression
Source: J Neuroinflammation. 2026 Jun 2;23:198. doi: 10.1186/s12974-026-03895-z (PMC13261964; doi:10.1186/s12974-026-03895-z)

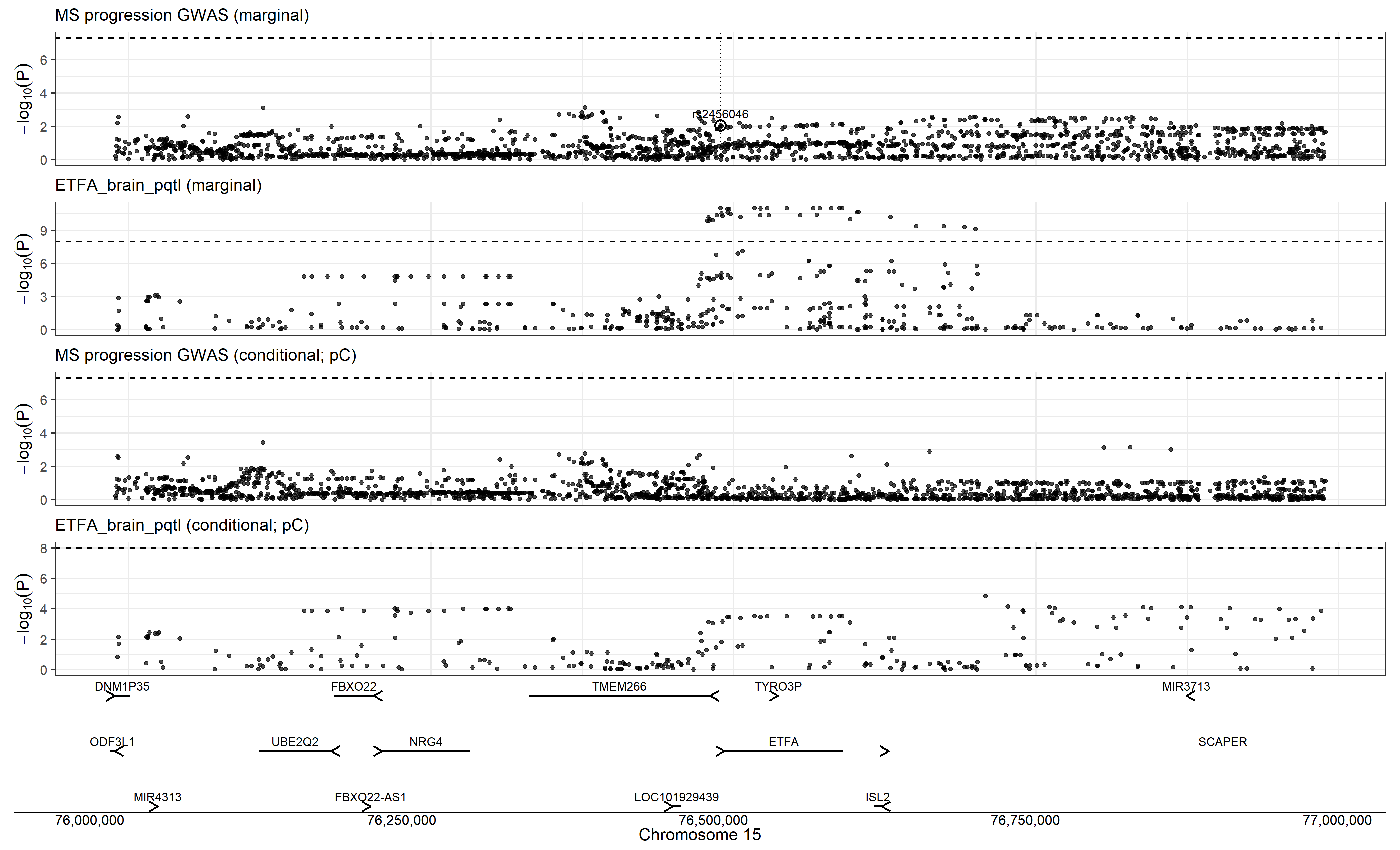

Supplement: Supplementary file 1 — Supplementary Material 1. [file 12974_2026_3895_MOESM1_ESM.png]

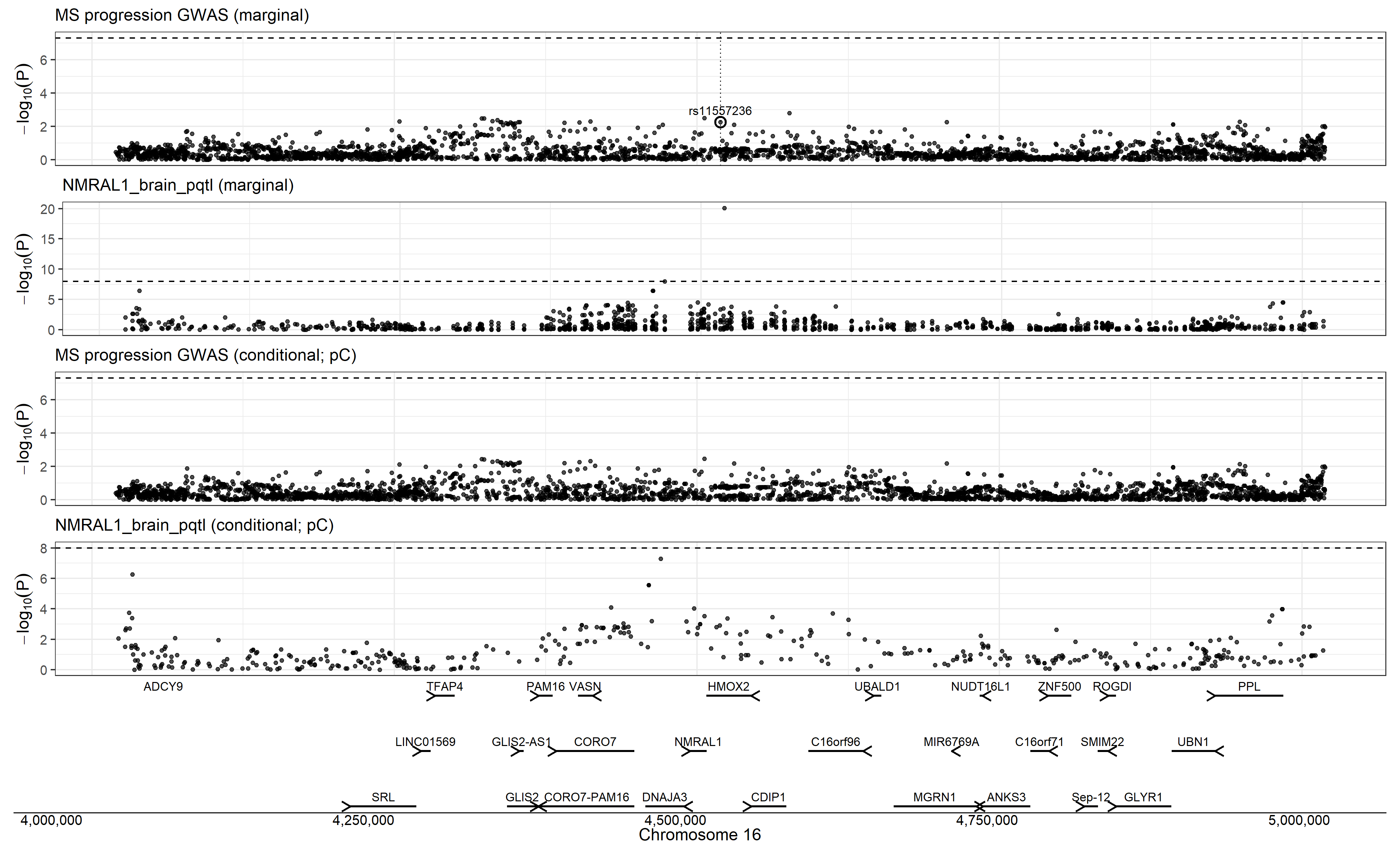

Supplement: Supplementary file 2 — Supplementary Material 2. [file 12974_2026_3895_MOESM2_ESM.png]

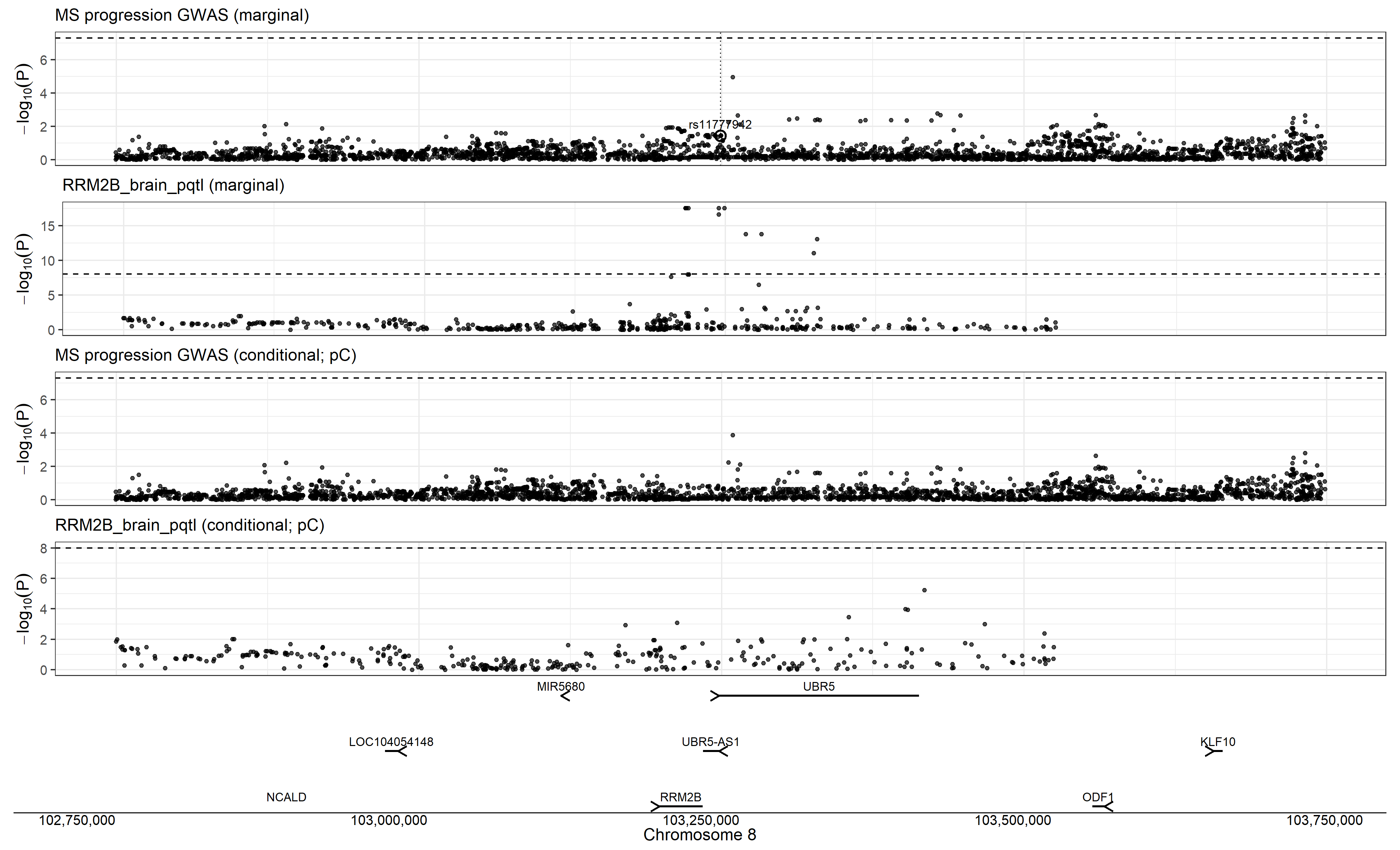

Supplement: Supplementary file 3 — Supplementary Material 3. [file 12974_2026_3895_MOESM3_ESM.png]

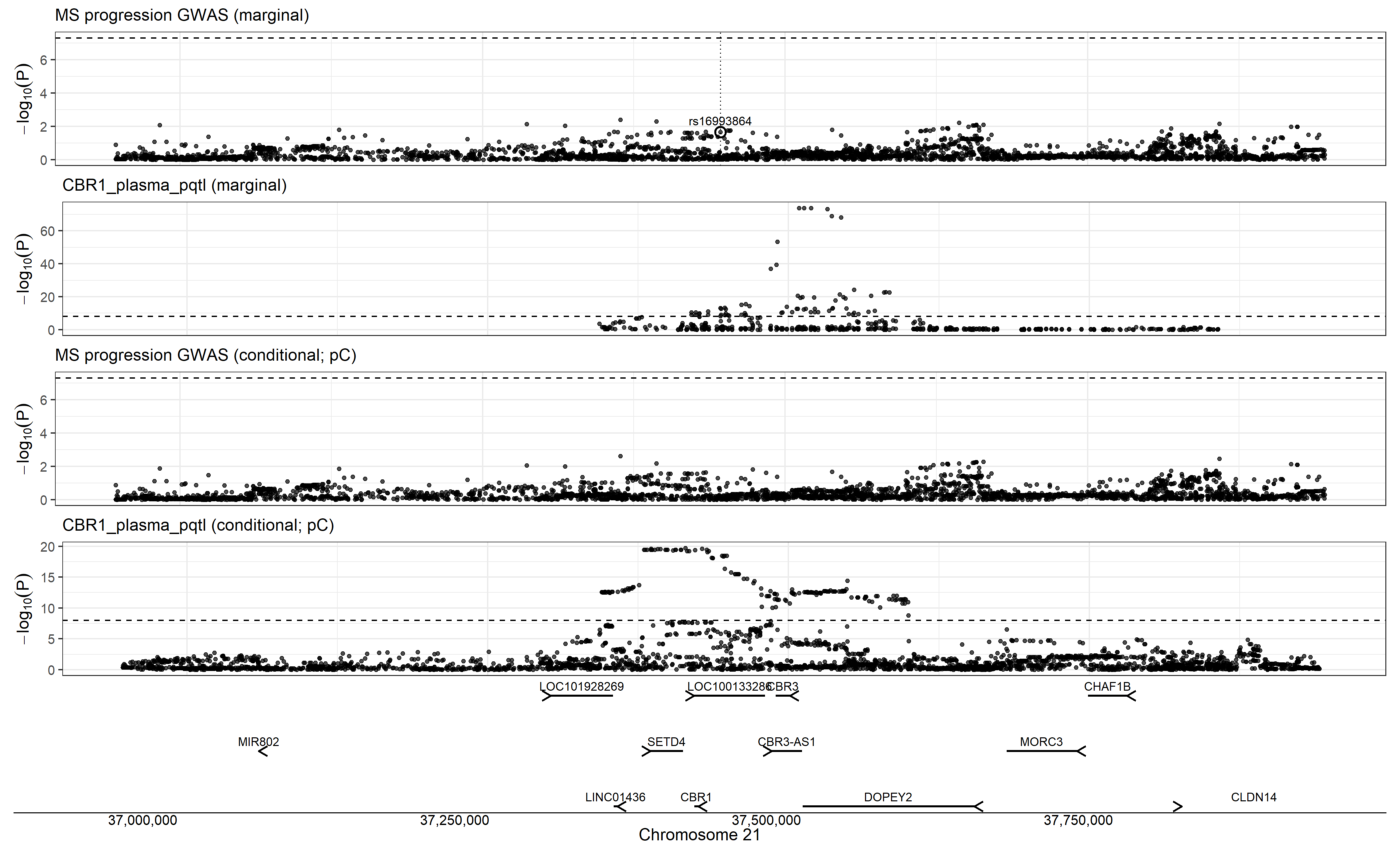

Supplement: Supplementary file 4 — Supplementary Material 4. [file 12974_2026_3895_MOESM4_ESM.png]

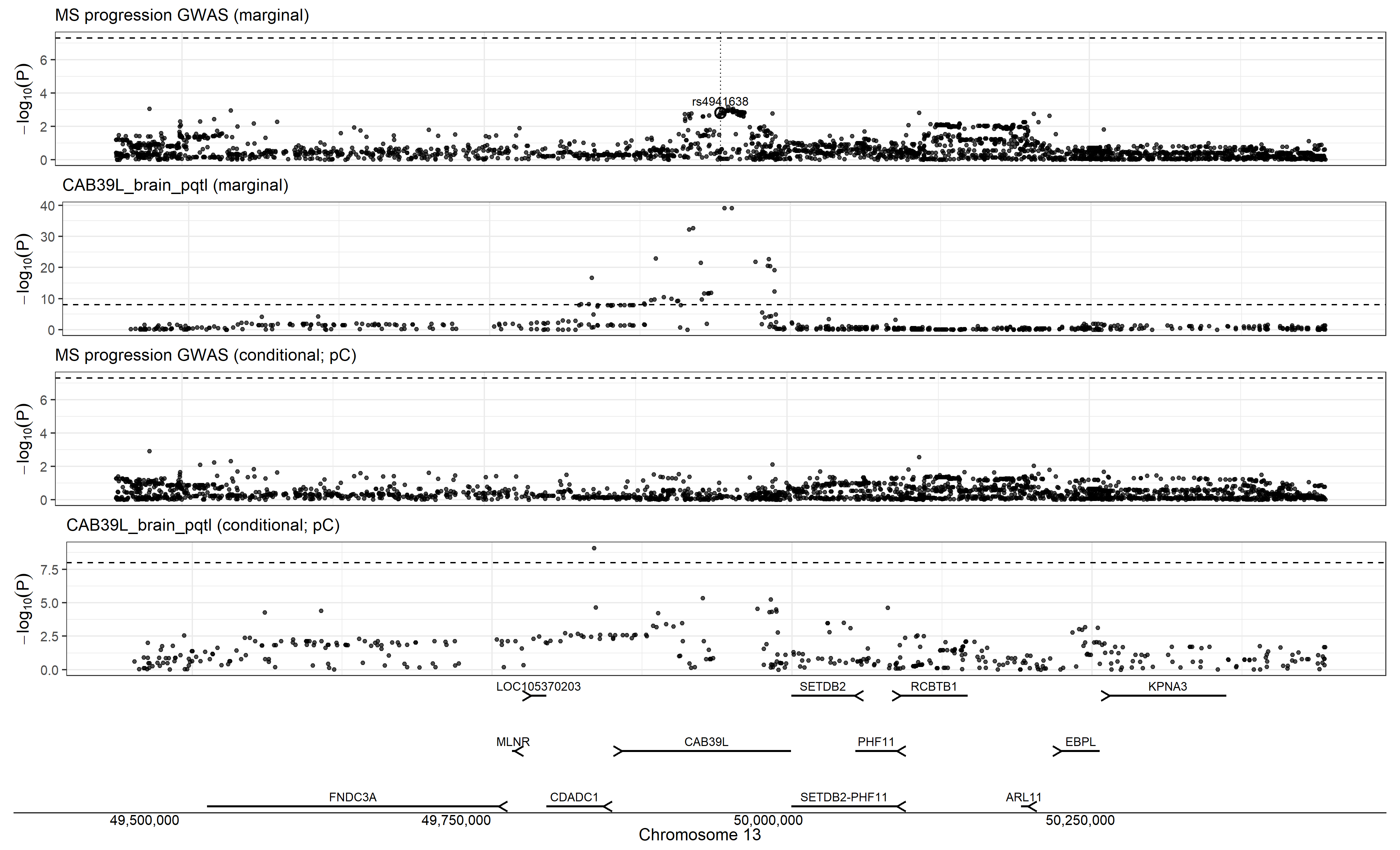

Supplement: Supplementary file 5 — Supplementary Material 5. [file 12974_2026_3895_MOESM5_ESM.png]

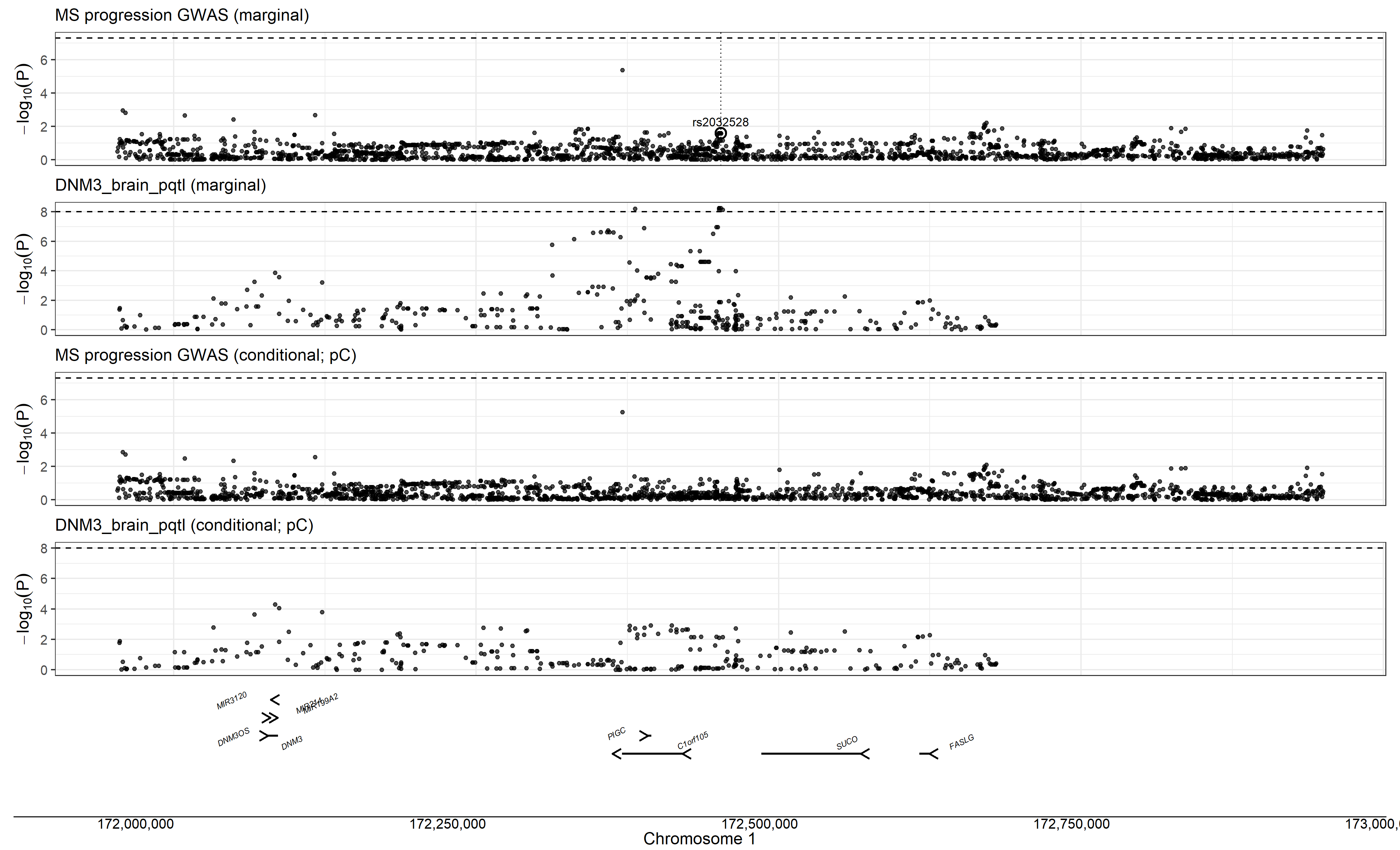

Supplement: Supplementary file 6 — Supplementary Material 6. [file 12974_2026_3895_MOESM6_ESM.png]
